# ^18^F-fluoro-2-deoxy-D-glucose positron emission tomography/computed tomography in the diagnosis and follow-up of primary hepatic diffuse large B-cell Lymphoma: A clinical case report

**DOI:** 10.1097/MD.0000000000018980

**Published:** 2020-01-31

**Authors:** Li Wang, Ping Dong, Weiming Hu, Bole Tian

**Affiliations:** a Department of Pancreatic Surgery; b Department of Nuclear Medicine, West China Hospital, Sichuan University, Chengdu, P.R. China.

**Keywords:** diffuse large B cell lymphoma, PET/CT, primary hepatic lymphoma, radiofrequency ablation

## Abstract

**Rationale:**

Primary hepatic lymphoma (PHL) is an extremely rare manifestation of extranodal non-Hodgkin lymphoma. There were few cases about PHL in recent years, while cases using positron emission tomography (PET) modalities for both diagnosis and follow-up were even rare.

**Patient concerns:**

A 29-year-old man complaining of dull epigastric pain for 2 weeks.

**Diagnosis:**

The features of liver biopsy and immunohistochemistry were consistent with diffuse large B cell lymphoma. Since there were no other foci of lymphoma on the ^18^F-fluoro-2-deoxy-D-glucose (^18^F**-**FDG) PET/computed tomography (CT) images, the patient was further diagnosed with PHL.

**Interventions:**

Since the lesions were mainly confined to the right lobe of liver, partial hepatectomy and radiofrequency ablation were performed. Subsequently, 6 cycles of rituximab, cyclophosphamide, adriamycin, vincristine, dexamethasone regimen were performed.

**Outcomes:**

The patient recovered well postoperatively and was discharged 1 week after surgery. Fortunately, the follow-up ^18^F**-**FDG PET/CT scan 36 months later revealed no abnormal FDG uptake, indicating the absence of relapse.

**Lessons:**

As the superiority in excluding other organ involvement, ^18^F**-**FDG PET/CT should be considered as the preferable imaging modality for the diagnosis and follow-up of PHL. Besides chemotherapy, surgical resection should be considered initially. If radical R0 resection could not be done, partial hepatectomy with radiofrequency ablation may also offer an appropriate alternative treatment.

## 1 Introduction

Primary hepatic lymphoma (PHL) is an extremely rare manifestation of extranodal non-Hodgkin lymphoma, which comprises only about 0.4% of all cases of extranodal non-Hodgkin lymphomas and only 0.016% of all non-Hodgkin lymphomas.^[1]^ Upon cases reported previously, the clinical manifestations and laboratory tests remains nonspecific.^[2]^ Indeed, the disease is usually misdiagnosed preoperatively. Liver biopsy with specific immunohistochemistry is the only test to confirm the diagnosis.^[2,3]^^18^F-fluoro-2-deoxy-glucose positron emission tomography/computed tomography (^18^F**-**FDG PET/CT) has been an important modality for detecting tumors, monitoring therapy in malignant cancers including lymphoma,^[4,5]^ while reports about the imaging characteristics of ^18^F**-**FDG PET/CT of patients with PHL are scarce. Herein, we report a case of PHL with ^18^F**-**FDG PET/CT images in the process of both diagnosis and follow-up.

## 2 Case presentation

A 29-year-old Chinese man was admitted to our hospital after 2 weeks of dull epigastric pain, without complaints of nausea, vomiting, or fever. On physical examination, he was afebrile (36.9°C), normotensive (114/74 mm Hg), without any signs of jaundice and in good spirit. There was no splenomegaly or enlarged superficial lymph nodes, and the liver was not palpable.

Ultrasound examination was performed first, and multiple abnormal hypoechoic lesions in liver were found. Laboratory results were within the normal range apart from decreased albumin of 34.9 g/L (reference range, 40.0–55.0 g/L) and elevated lactate dehydrogenase of 766.0 U/L (reference range, 140.0–271.0 U/L). Antinuclear antibody and rheumatoid factor, anti-smooth muscle and anti-mitochondrial antibodies were negative. Carcino-embryonic antigen (CEA), carbohydrate antigen 19-9 (CA19-9), and alpha-fetoprotein (AFP) were normal.

**Figure 1:**
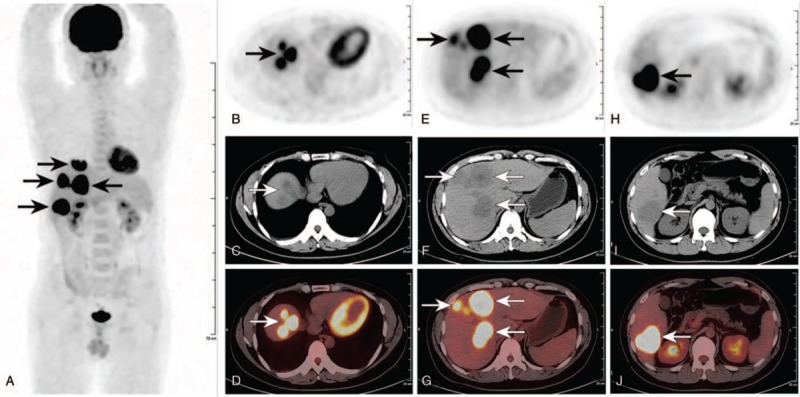
The preoperative ^18^F-FDG PET/CT images. (A) The MIP image showed multiple FDG-avid lesions (black arrows) confined to the liver without abnormal uptake in any other tissues or organs. (B–J) Figures displayed from upper to lower axial ^18^F-FDG PET, CT and PET/CT fusion, respectively. These images revealed multiple well-defined intense FDG-avid lesions mainly involved the right lobe and partial left medial segments of the liver (B–J, black and white arrows). Among these FDG-avid lesions, the maximum lesion located in segment VI with the diameter of 49 mm and SUVmax of 24.9 (H–J, black and white arrows). ^18^F-FDG = ^18^F-fluoro-2-deoxy-D-glucose, MIP = maximum intensity projection, PET/CT = positron emission tomography/computed tomography, SUVmax = maximum standardized uptake value.

Since hepatic metastases could not be excluded initially, the patient was administered with ^18^F-FDG (403.7 MBq, 5 MBq/kg body weight) and imaged for 2.5 minutes per bed after approximately 1 hour ^18^F-FDG injection on a Gemini 16 PET/CT scanner (Philips Healthcare, the Netherlands) to identify potential primary tumor. As the FDG PET/CT images demonstrated, multiple well-defined intense FDG-avid lesions mainly involved the right lobe and partial left medial segments of the liver (Fig. 1A–J, black and white arrows) without abnormal uptake in any other tissues or organs. Among these FDG-avid lesions, the maximum lesion located in segment VI with the diameter of 49 mm and maximum standard uptake value (SUVmax) of 24.9 (Fig. 1H–J, black and white arrows). Considering the findings of the FDG PET/CT, the possibility of primary hepatic tumor was raised.

Subsequently, the patient was performed with open laparotomy for diagnosis and treatment. There was neither obvious ascites in abdominal cavity, nor significant mesenteric or retroperitoneal lymphadenopathy. As the intraoperative ultrasound revealed, the tumors were located in segments IV, V, VI, VII, and VIII. Subsequently, partial hepatectomy and radiofrequency ablation were performed. Liver biopsy showed heavy infiltration mainly of large lymphoid cells. Immunohistochemical staining was positive for CD20, LCA, CD10, bcl-6, and mum-1, negative for CD3, PCK, and TdT, and the proliferation index was very high with 95% of the cells Ki-67 positive, indicating a diagnosis of diffuse large B-cell lymphoma (DLBCL) that originated in the liver, since there were no other foci of lymphoma. According to the Lugano classification which was adopted by the American Joint Committee on Cancer 8th staging manual,^[6,7]^ the present case was further considered with stage IV disease.

**Figure 2:**
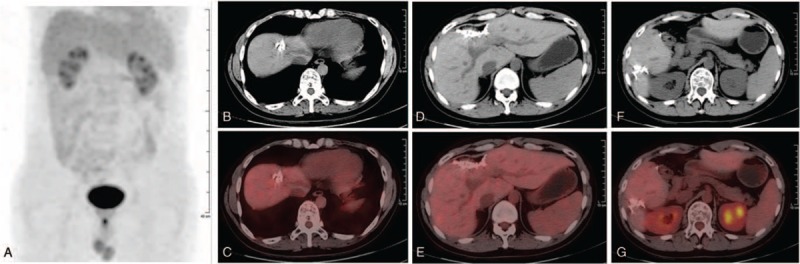
The follow-up ^18^F-FDG PET/CT images 3 yr after treatment. Both the MIP (A) and axial images (CT: B, D, and F; PET/CT fusion: C, E, and G) showed no abnormal uptake in the abdomen. ^18^F-FDG = ^18^F-fluoro-2-deoxy-D-glucose, MIP = maximum intensity projection, PET/CT = positron emission tomography/computed tomography.

The patient was discharged 1 week postoperatively, and received 6 cycles of rituximab, cyclophosphamide, adriamycin, vincristine, dexamethasone regimen 1 month after surgery. Following the completion of these therapies, contrast-enhanced CT revealed absence of any suspicious lesion. Besides, 36 months later, the follow-up FDG PET/CT scan (398.9 MBq) also showed no abnormal uptake in the abdomen (Fig. 2A–G), indicating score 1 and complete remission (CR) according to the Lugano classification lymphoma response criteria.^[7]^

We have obtained the patient's informed consent for the publication of this case.

## 3 Discussion

DLBCL is a kind of common lymphoma and accounts for approximately 25% of all non-Hodgkin lymphomas in the developed world.^[8]^ In up to 40% of cases, the disease arises in extranodal and extramedullary tissues, with the stomach/gastrointestinal tract as the most common site.^[9]^ However, the disease arises in the liver, namely PHL, was extremely rare.^[1,10]^ To the best of our knowledge, there were few cases of PHL had been presented in the recent years,^[11–13]^ among which using FDG PET/CT as the imaging modality for both diagnosis and follow-up were even rare.

As a notably rare subtype of non-Hodgkin lymphoma, PHL is defined as lymphoma confined to the liver with no evidence of lymphomatous involvement in the spleen, lymph nodes, bone marrow, or other lymphoid structures.^[14]^ The diagnosis of non-Hodgkin lymphoma could be confirmed by liver biopsy and immunohistochemistry. While, as stated in the definition above, the diagnosis of PHL require excluding any other lymphomatous involvement beyond liver, and this excluding largely relies on imaging modalities. Based only on morphological features, it is difficult for traditional imaging modalities to identify all the small malignant lesions within the whole body. Whereas, based on both morphological and glucose metabolic features of malignant tumors, FDG PET/CT is an excellent noninvasive imaging modality with high sensitivity and specificity in the detection of lymphoma in the extent of whole body.^[15]^ As revealed by the preoperative FDG PET/CT images of the present patient, multiple well-defined intense FDG-avid lesions with a SUVmax of 24.9 were detected 2 weeks after the onset of symptom, which contribute to the relatively early diagnosis and preferable treatment of the patient.

Besides early detection of occult lymphomas, the prognostic value of ^18^F**-**FDG PET/CT in multiple types of lymphoma had also been reported. As reported by Cottereau et al,^[16]^ combining metabolic tumor volume obtained from ^18^F**-**FDG PET/CT with a parameter reflecting the tumor burden dissemination further improves DLBCL patient risk stratification at staging. Recently, a prospective multicenter international trial (NCT00921414) of mantle cell lymphoma found that SUVmax > 10.3 was associated with aggressive variants (Ki67 > 30%), and both significant shorter progression free and overall survival.^[17]^ Meanwhile, the metabolic response reflected by PET/CT following therapies was also reported to be practical in predicting survival of patients with lymphomas.^[18–20]^ So far; however, mostly due to the rarity of it, the prognostic value of ^18^F**-**FDG PET/CT in patients with PHL has rarely been described. Indeed, even the clinical manifestations and laboratory tests of it still remains nonspecific.^[2]^

Also, the treatment of patients with PHL reported in the literature has not been consistent. Systemic combination chemotherapy was used as the main modality of treatment, which could meet a CR rate of 83.3%.^[10]^ Whereas, 30% to 40% of patients treated with chemotherapy alone cannot be cured and consequently suffer relapse.^[2]^ Surgical resection remains the chance for cure for patients with localized lesions.^[21]^ For DLBCL, which is the predominant histology of PHL, the 5-year progression-free survival could reach 80% to 85% in patients at a limited stage, while patients with advanced disease show a dramatic decrease of 5-year progression-free survival down to 50%.^[22]^ Thus, early detection and preferable treatments could bring a much better outcome. In the present case, after the detection of multiple intense FDG-avid lesions confined to the liver, partial hepatectomy and radiofrequency ablation were performed before chemotherapy. In the follow-up 36 months after surgery, the patient lived well with no relapse which indicating the success of treatment.

## 4 Conclusion

In conclusion, as a rare disease, PHL should be considered in any patient with multiple lesions of the liver and with normal AFP, CA19-9, and CEA. Besides liver biopsy, FDG PET/CT should also be considered in these patients in order to exclude any other organ involvement and further confirm the “primary hepatic” part of the diagnosis of PHL. Meanwhile, PET modalities should also be considered in the follow-up of these patients. Furthermore, as a large portion of patients with chemotherapy alone results in unfavorable outcomes, surgical intervention should be considered initially for patients with PHL. When R0 resection could not be reached, partial hepatectomy combined with radiofrequency ablation may also achieve preferable outcome.

## Author contributions

**Conceptualization:** Li Wang, Ping Dong, Bole Tian.

**Investigation:** Ping Dong.

**Methodology:** Ping Dong, Weiming Hu.

**Software:** Ping Dong.

**Supervision:** Li Wang, Bole Tian.

**Writing – original draft:** Li Wang.

**Writing – review and editing:** Bole Tian.
